# A mechanistic role for the chromatin modulator, NAP1L1, in pancreatic neuroendocrine neoplasm proliferation and metastases

**DOI:** 10.1186/1756-8935-7-15

**Published:** 2014-07-21

**Authors:** Simon Schimmack, Andrew Taylor, Ben Lawrence, Daniele Alaimo, Hubertus Schmitz-Winnenthal, Markus W Büchler, Irvin M Modlin, Mark Kidd

**Affiliations:** 1 Gastrointestinal Pathobiology Research Group, Yale University School of Medicine, PO Box 208602, New Haven, CT 06510, USA; 2 Department of General, Visceral and Transplantation Surgery, University Hospital Heidelberg, Im Neuenheimer Feld 110, Heidelberg 69120, Germany

**Keywords:** NAP1L1, Pancreatic neuroendocrine neoplasms, pNETs, Promoter methylation, p57, Proliferation

## Abstract

**Background:**

The chromatin remodeler NAP1L1, which is upregulated in small intestinal neuroendocrine neoplasms (NENs), has been implicated in cell cycle progression. As p57^Kip2^ (CDKN1C), a negative regulator of proliferation and a tumor suppressor, is controlled by members of the NAP1 family, we tested the hypothesis that NAP1L1 may have a mechanistic role in regulating pancreatic NEN proliferation through regulation of p57^Kip2^.

**Results:**

NAP1L1 silencing (siRNA and shRNA/lipofectamine approach) decreased proliferation through inhibition of mechanistic (mammalian) target of rapamycin pathway proteins and their phosphorylation (*p* < 0.05) in the pancreatic neuroendocrine neoplasm cell line BON in vitro (*p* < 0.0001) and resulted in significantly smaller (*p* < 0.05) and lighter (*p* < 0.05) tumors in the orthotopic pancreatic NEN mouse model. Methylation of the *p57*^*Kip2*^ promoter was decreased by NAP1L1 silencing (*p* < 0.05), and expression of p57^Kip2^ (transcript and protein) was upregulated. For methylation of the *p57*^*Kip2*^ promoter, NAP1L1 bound directly to the promoter (−164 to +21, chromatin immunoprecipitation). In 43 pancreatic NEN samples (38 primaries and 5 metastasis), NAP1L1 was over-expressed in metastasis (*p* < 0.001), expression which was inversely correlated with p57^Kip2^ (*p* < 0.01) on mRNA and protein levels. Menin was not differentially expressed.

**Conclusion:**

NAP1L1 is over-expressed in pancreatic neuroendocrine neoplasm metastases and epigenetically promotes cell proliferation through regulation of *p57*^*Kip2*^ promoter methylation.

## Background

Pancreatic neuroendocrine neoplasms (pNENs) are an aggressive form of cancer that are derived from a heterogeneous population of endocrine cells [1]. While approximately 5% of tumors exhibit germ-line mutations in MEN-1, VHL, NF-1, or TSC [2], the pathogenesis of the majority of lesions remains ill-understood [3]. Recently, sequencing of sporadic pNENs identified mutations in chromatin remodeling genes including menin (in 44% of the cases) and DAXX/ATRX (43%) [4]. The epigenome therefore is a potential target in the etiopathology of pNENs.

One gene that was not mutated was nucleosome assembly protein 1-like 1 (*NAP1L1*). NAP1L1 belongs to a family which is thought to be involved in nucleosome assembly and exchange of histone H2A-H2B dimmers [5-7], as well as transcriptional regulation and cell cycle progression [8]. Inactivation of NAP1 significantly alters gene expression profiles [9], while deletion of NAP1L2 results in embryonic lethality [10]. NAP1L2, NAP1L3, and NAP1L5 [11-14] are neuron-specific and play a role in neuronal differentiation, proliferation, and apoptosis [15]. NAP1-like proteins have similar activities regarding nucleosome assembling; NAP1L1, however, displays the highest disassembly activity [16]. Increased expression of NAP1L1 may be related to cell growth, as levels of both NAP1L1 mRNA and protein increase rapidly in conjunction with cell proliferation in a T-lymphoid cell model [17]. NAP1L1 is also over-expressed in fetal liver compared with adult liver [18], in hepatoblastoma compared to healthy adult liver [19], and in 50% of colon cancer [20]. In addition, NAP1L1 is elevated in malignant adenocarcinoids compared to normal mucosa [21] and has been reported to be over-expressed in small intestinal NENs [22]. Little, however, is known regarding its expression and potential role in the pathogenesis of pNENs.

The regulation of cell cycle inhibitors such as the CDK inhibitor p57^Kip2^ (CDKN1C) is controlled by members of the NAP1 family [15], suggesting one mechanism by which NAP1L1 could regulate proliferation. P57^Kip2^ is highly expressed in G_0_/G_1_ and decreases during progression through G_1_ − S simultaneously with the activation of cyclin-CDK complexes [23,24]. Accordingly, over-expression of p57^Kip2^ induces G_1_ arrest in cultured cells [25]. In pancreatic adenocarcinomas, p57^Kip2^ protein is decreased in >50% of lesions which correlates with more aggressive forms [26,27]. In endocrine neoplasms, p57^Kip2^ expression was absent in 75% of malignant adrenocortical tumors [28], and in a second study in 47 adrenal tissues, no p57^Kip2^ mutations were evident but a decreased expression was detected in malignant tumors [29].

We hypothesized that NAP1L1 played a role in regulating cell entry into the S-phase through transcriptional regulation of factors that controlled neuroendocrine cell growth and proliferation. We considered that p57^Kip2^, as a tumor suppressor, would be silenced in proliferating/neoplastic neuroendocrine cells as a consequence of NAP1L1 activity. Since one of the major inherited genes involved in pancreatic NENs is menin, a known growth inhibitor and chromatin remodeler [30,31], we also examined its expression to assess whether it was associated with NAP1L1 and p57^Kip2^. We used a dataset of well-characterized pancreatic NENs (Heidelberg, Yale collections [32]) as well as the BON cell line [32,33] as a model to examine the function of NAP1L1.

## Results

### NAP1L1 knockdown and tumor cell signaling in vitro

**Figure 1:**
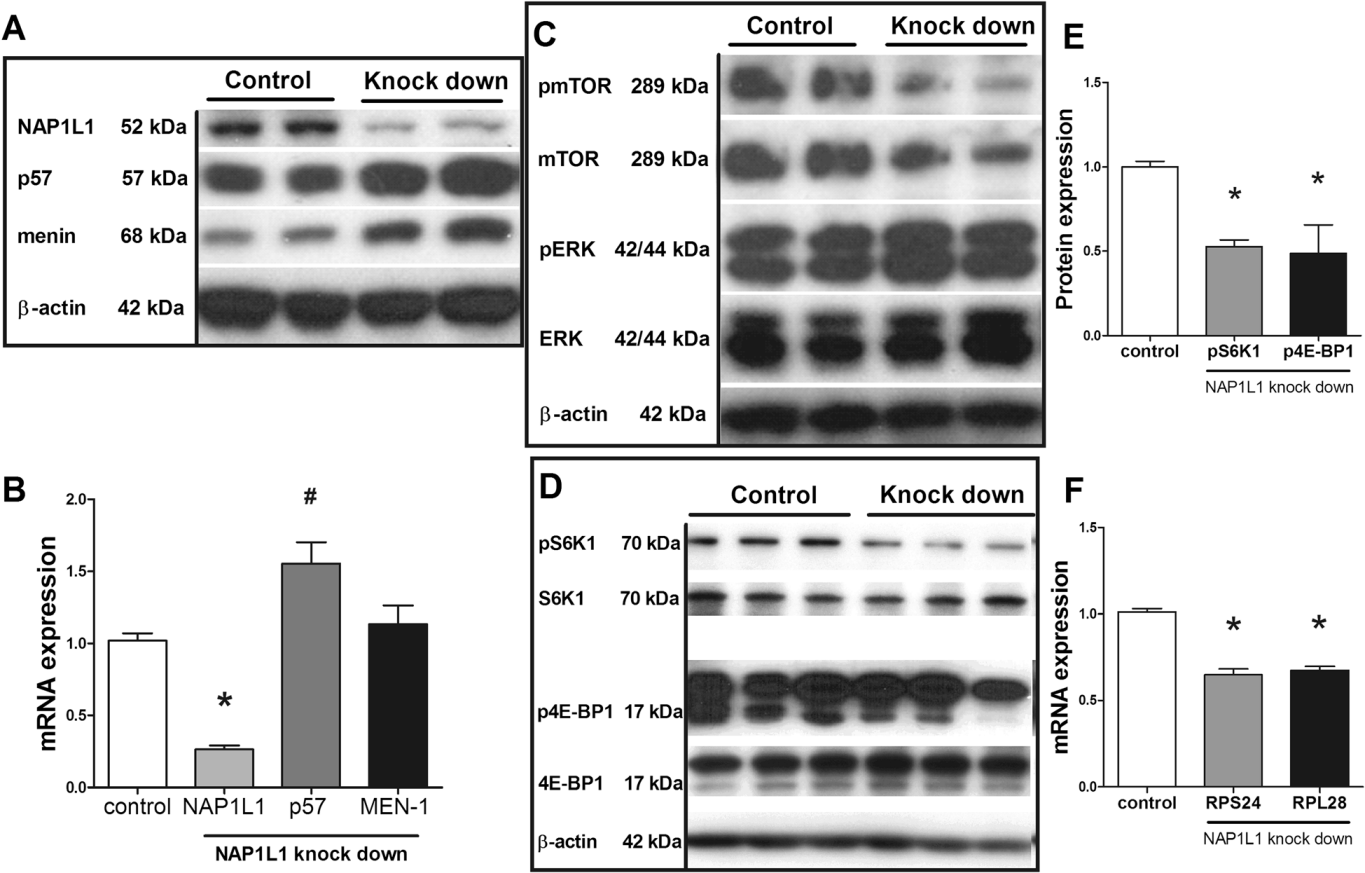
**Effect of NAP1L1 silencing.** Effect of NAP1L1 silencing on p57^Kip2^ and menin expression as well as on the mTOR pathway in BON cells. An effective NAP1L1 knockdown (approximately 3-fold, *p < 0.001) increased *p57*^*Kip2*^ protein **(A)** and mRNA **(B)** (#*p* < 0.01), while menin was increased at protein level only **(A and B)**. The ERK pathway was unaffected in NAP1L1-silenced cells, while total mTOR as well as phosphorylated mTOR decreased in NAP1L1 silenced BON cells **(C)**. The mTOR downstream effectors eukaryotic initiation factor 4E-binding protein (4E-BP1) and S6 kinase (S6K1) also showed less protein expression and decreased phosphorylation when NAP1L1 was absent **(D and E)** (*p < 0.05). MRNA levels of the two ribosomal proteins RPS24 and RPL28, as a measure of mTOR activity, were also decreased in NAP1L1 knockdown cells (*p < 0.05), indicating that NAP1L1 may promote mTOR signaling **(F)**. 4E-BP1, lower band. Mean ± standard error of mean (SEM).

Initially, we examined the effect of NAP1L1 silencing in the BON cell line. NAP1L1 was silenced 3-fold (*p* < 0.001) as demonstrated at both protein (Figure 1A) and mRNA (Figure 1B) levels. This was associated with an increase in p57^Kip2^ mRNA (*p* < 0.01) and protein. While *menin* mRNA did not change, protein increased in NAP1L1-silenced cells. Growth signaling pathways were also altered by NAP1L1 silencing. Specifically, mechanistic (mammalian) target of rapamycin (mTOR) and phosphorylated mTOR was reduced in NAP1L1-silenced down cells (Figure 1C). Consequently, downstream effectors such as eukaryotic initiation factor 4E-binding protein (4E-BP1) as well as S6 kinase (S6K1), which mTOR regulates through phosphorylation [34], were reduced (Figure 1D,E, *p* < 0.05). Additionally, mRNA expression of ribosomal proteins including S24 (RPS24) and ribosomal protein L28 (RPL28) were also decreased as expected [35] when NAP1L1 was silenced (Figure 1F, *p* < 0.05). The ERK pathway was unaffected (Figure 1B). This suggests that NAP1L1 may promote proliferation via mTOR signaling.

### NAP1L1 and tumor cell proliferation in vitro

**Figure 2:**
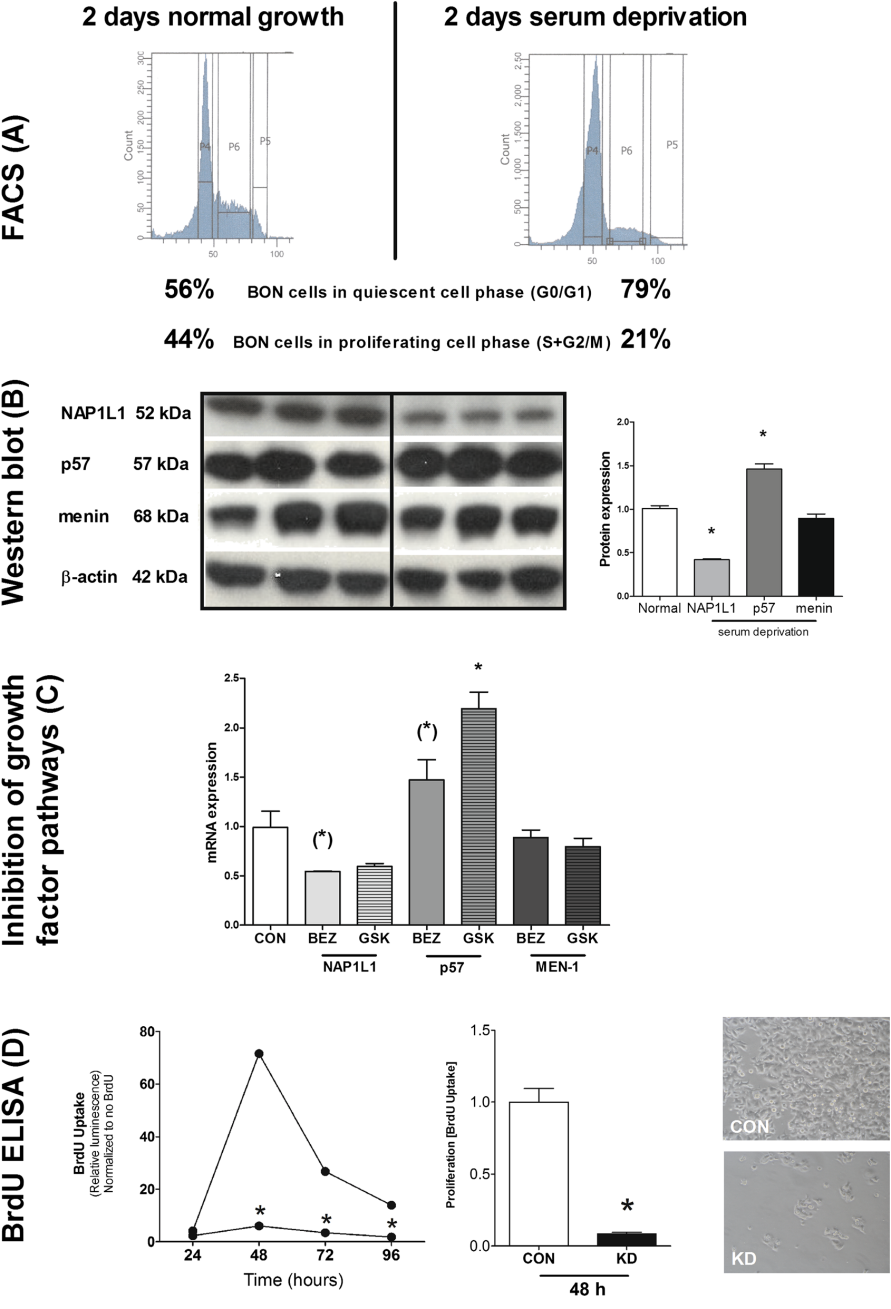
**The effect of NAP1L1 on tumor cell proliferation *in vitro.*** In proliferating BON cells, 56% were in the quiescent cell phase (G_0_/G_1_) and 44% in the proliferating cell phase (S and G_2_/M). After two days of serum deprivation, 80% of the cells were quiescent **(A)**. A decrease of NAP1L1 (*p < 0.05) and an increase of p57^Kip2^ (*p < 0.05) protein expression were evident **(B)**. Inhibiting proliferation by targeting the mTOR pathway (BEZ235) and ERK pathway (GSK1120212) resulted in a similar pattern of expression: NAP1L1 tended to be decreased ((*) *p* = 0.06) and p57^Kip2^ was increased (*p < 0.05) while menin did not alter **(C)**. BON cells without NAP1L1 did not grow (*p < 0.0001) and exhibited decreased cell numbers **(D)**. Mean ± SEM.

To evaluate the expression of NAP1L1, p57^Kip2^, and menin during cell proliferation, we first examined protein expression during the different phases of the cell cycle (Figure 2A). In proliferating BON cells (2 days of growth in normal media), quiescent (G_0_/G_1_) and cycling (S and G_2_/M) cell populations were evenly divided. The shift to quiescence by 2 days of serum deprivation (80% of the cells were in G_0_/G_1_ phase, Figure 2A) was associated with decreased NAP1L1 (*p* < 0.05) and increased P57^Kip2^ protein expression (*p* < 0.05) (Figure 2B); menin was not increased (Figure 2B). Pharmaceutical inhibition of growth factor signaling pathways with either BEZ235 (targeting mTOR) or GSK1120212 (targeting ERK) generated similar alterations. Inhibition of these growth factor signaling pathways tended to decreased *NAP1L1* (*p* = 0.06) and concomitantly increased *p57*^*Kip2*^ mRNA expression (*p* < 0.05), while *menin* did not alter (Figure 2C).

To investigate a role for NAP1L1 in tumor proliferation, the effect of NAP1L1 knockdown in BON cells was evaluated in BrdU incorporation assays. Cells were studied over a 4-day period. By 48 h, there was a significant elevation in BrdU uptake in non-silenced/normal BON cells compared to NAP1L1-silenced BON cells (Figure 2D, *p* < 0.0001, approximately 40-fold) as well as a marked decrease in the number of cells in these experiments (Figure 2D).

### NAP1L1 and tumor cell proliferation *in vivo*

**Figure 3:**
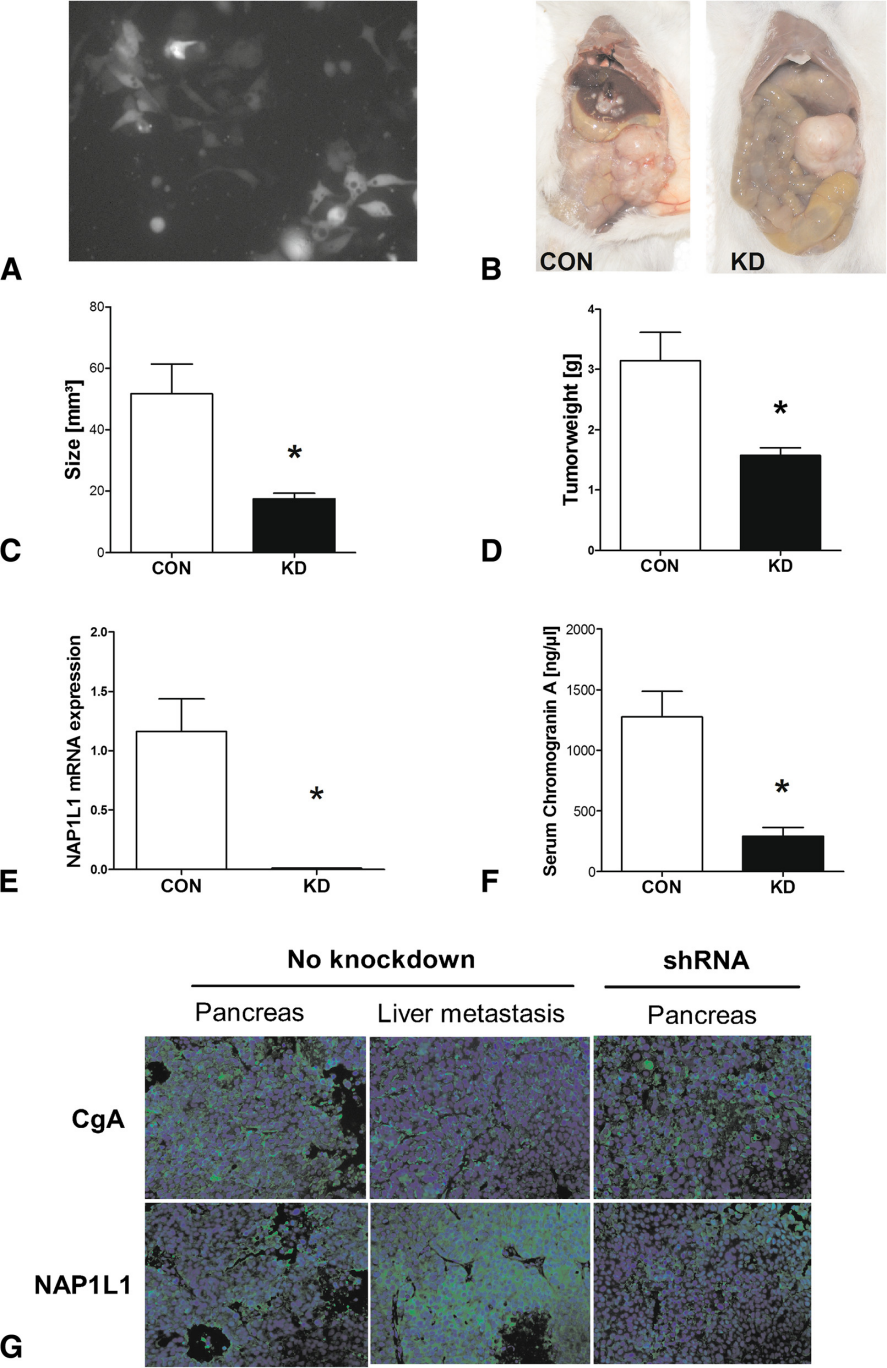
**Impact of NAP1L1 silencing of tumor growth in an orthotopic pNEN mouse model.** After having established stable NAP1L1 knockdown BON cells (confirmation of shRNA internalization by fluorescence microscopy **(A)** and thereafter puromycin-selection), we injected 1.5 × 10^6^ transfected cells in 100 μl PBS into the pancreas tail of nine Ns/J-mice by flank-laparotomy (CON = 4 mice, KD = 5 mice). Six weeks after pancreas injection, necroscopy identify large tumors with metastases in control injected mice **(B)**, while KD-injected mice had smaller **(C)** (*p < 0.05) and lighter **(D)** (*p < 0.05) tumors that were all localized. KD tumors expressed less NAP1L1 than controls **(E and G)**. Circulating chromogranin A (CgA) levels were also decreased in serum of KD mice **(F)** (*p < 0.05), and expression of this marker was lower in the liver metastases **(G)**. Mean ± SEM.

We next examined whether NAP1L1-silencing reduced tumor formation in an orthotopic pNEN mouse model. Transfection with shRNA was associated with a 70% internalization of the plasmid by fluorescence microscopy (Figure 3A). Three weeks after BON cell implantation (1.5 × 10^6^ cells, viability approximately 90%) into the pancreas tail at laparotomy, all mice in the control group (*n* = 4) had developed palpable tumors on the left upper side of the stomach. In contrast, only two of the five mice, injected with NAP1L1 knockdown cells, had tumors at 4 weeks. Both were small tactile tumors. After 6 weeks, we euthanized all nine mice (four with injection of control BON cells and five with injection of NAP1L1 knockdown BON cells) and found liver metastases in two of the four mice in the control group; all four mice had additional peritoneal metastases. None of the knockdown group exhibited any macroscopic evidence of metastases (Figure 3B). Pancreatic tumors in the control group were larger (51.75 ± 19.23 mm^3^) and heavier (wet weight, 3.14 ± 0.95 g) in comparison to the NAP1L1 knockdown group (17.48 ± 4.12 mm^3^ and 1.57 ± 0.29 g, *p* < 0.05) (Figure 3C,D). At an mRNA level, a decreased *NAP1L1* expression in knockdown tumors (*p* < 0.05) was confirmed (Figure 3E). Blood samples demonstrated a lower chromogranin A (CgA) serum expression in mice which had been injected with NAP1L1-knockdown cells (Figure 3F, *p* < 0.05) suggesting that CgA may, as in humans [36], be a marker of tumor burden. Its expression also correlated with tumor size (*r* = 0.88, *p* < 0.01) and weight (*r* = 0.9, *p* < 0.01) (*data not shown*). Histopathologically, a decrease in NAP1L1 expression was noted in knockdown tumors while the protein was highly expressed in liver metastases (Figure 3G). Expression of CgA was similar in normal and NAP1L1 knockdown tumors; metastases expressed less CgA (Figure 3G).

### NAP1L1 controls *p57*^*Kip2*^ expression by direct binding to its promoter

**Figure 4:**
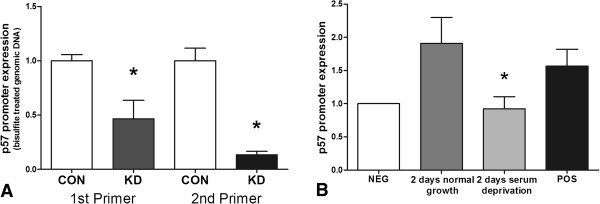
***p57***^***Kip2 ***^**promoter methylation and NAP1L1 binding.** Genomic DNA of normal BON cells and NAP1L1 silenced BON cells were treated with bisulfate, which converted cytosine of *unmethylated p57*^*Kip2*^ promoter into uracil. Methylation-specific polymerase chain reaction (MSPCR) was performed using two *p57*^*Kip2*^ promoter primers (forward first primer, CGCCAATCGCCGTGGTGTTG; forward second primer, ACTACATTATGCTAATCGCG; reverse primer, GCCAGGCCTGAGCGAGCGAG). Unmethylated *p57*^*Kip2*^ promoter resulted in low PCR products as demonstrated for NAP1L1-silenced cells **(A)** (*p < 0.05) indicating that NAP1L1 controls *p57*^*Kip2*^ expression by methylation of its promoter. In chromatin immunoprecipitation (ChIP) experiments, using the Pierce Agarose ChIP Kit (Thermo Scientific) according to the manufacturer’s instructions, a direct binding of NAP1L1 protein to the *p57*^*Kip2*^ promoter was evident in normal growing BON cells; non-proliferating cells did not silence the *p57*^*Kip2*^ promoter **(B)** (*p < 0.05). The calculated promoter expression in NAP1L1-bound DNA fragments is shown in relation to the control. Mean ± SEM.

To investigate mechanisms by which NAP1L1 regulated proliferation, we examined the methylation status of the *p57*^*Kip2*^ promoter region in normal and NAP1L1-silenced BON cells. As demonstrated (Figure 4A), a highly methylated (=silenced *p57*^*Kip2*^) promoter was detected in untreated BON cells. In contrast, the promoter was significantly less methylated (*p* < 0.05) in NAP1L1-silenced cells which suggested that NAP1L1 may be necessary for the maintenance of *p57*^*Kip2*^ promoter methylation and therefore inhibition of *p57*^*Kip2*^ transcription. We used ChIP analysis to examine whether NAP1L1 directly interacted with the *p57*^*Kip2*^ promoter. In normal growing BON cells, NAP1L1-bound DNA fragments contained the *p57*^*Kip2*^ promoter region (−164 to +21) (Figure 4B). In low-proliferating BON cells (2-day serum deprivation), no binding of NAP1L1 to the *p57*^*Kip2*^ promoter was detected. We interpret these results to provide an explanation for the increased p57^Kip2^ protein expression (no NAP1L1 binding and therefore inhibition, coupled to activation through known *p57*^*Kip2*^ transcriptional regulators, e.g., HDACI/II) [37] in serum-deprived BON cells (Figure 2B).

### Screening of NAP1L1, p57^Kip2^, and menin expression in normal and neoplastic pancreas

**Figure 5:**
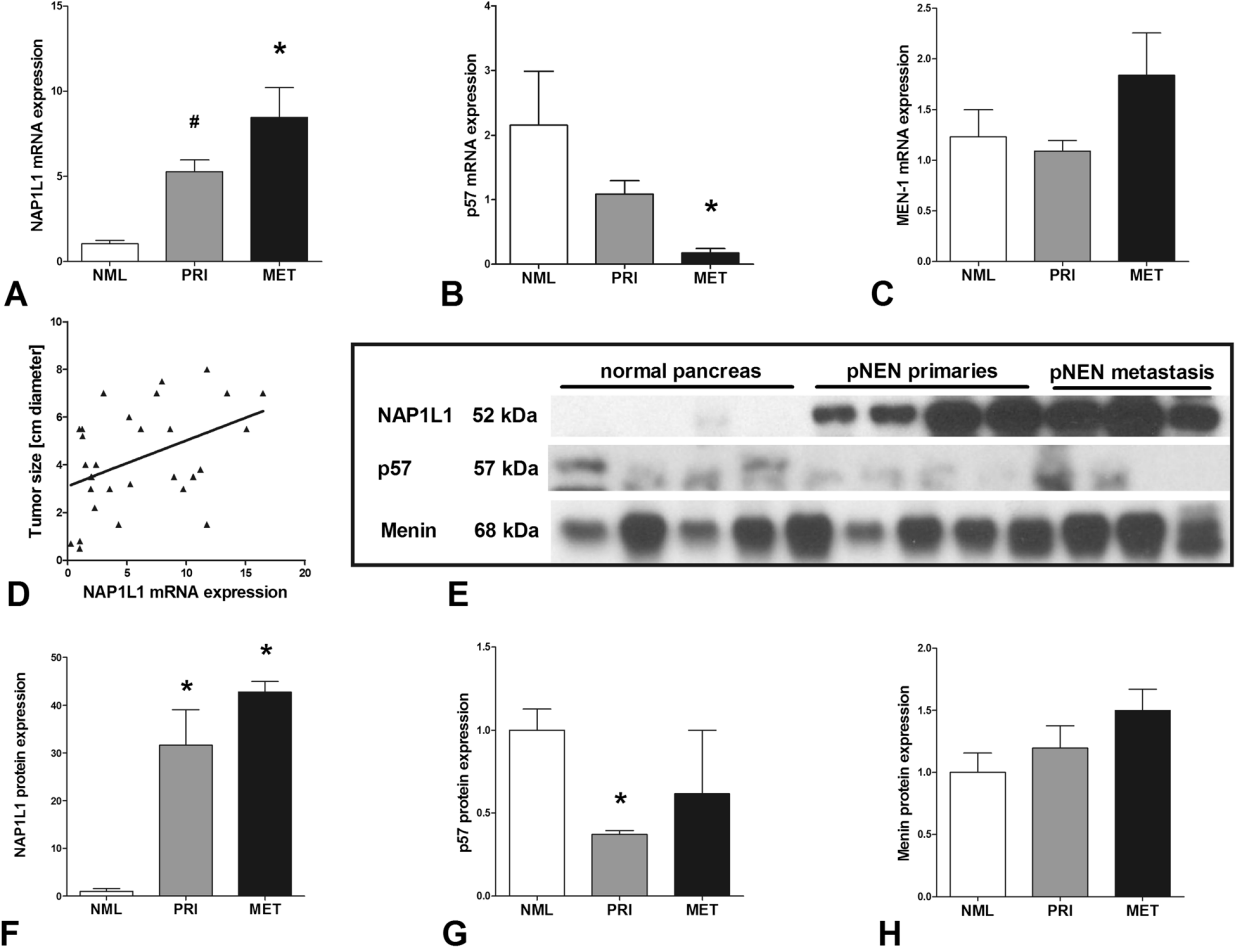
**NAP1L1, p57**^**Kip2**^**, and menin expression in pancreatic neuroendocrine neoplasms compared to normal pancreatic tissue.** NAP1L1 was increased at mRNA level **(A)** (Kruskal-Wallis *p* < 0.001) in pNEN primaries (#*p* < 0.01) and metastases (*p < 0.01) and expression correlated with tumor size **(D)** (*r* = 0.42, *p* < 0.05). In contrast, *p57*^*Kip2*^ mRNA was downregulated in pNEN metastases **(B)** (*p < 0.01). Menin was not differently expressed in pNEN in comparison to normal tissue in this sample set **(C and H)**. Western blot analysis **(E)** confirmed elevated NAP1L1 protein levels **(F)** (*p < 0.05) and downregulated expression of p57^Kip2^ in pNENs **(G)** (*p < 0.05). Mean ± SEM.

To evaluate whether the *in vitro* and *in vivo* mechanistic observations were clinically relevant, we screened NAP1L1, p57^Kip2^, and menin in ten normal pancreas and 43 pNEN samples. *NAP1L1* mRNA was over-expressed in pNEN primaries (*p* < 0.01) and pNEN metastasis (*p* < 0.01) compared to normal pancreas (Figure 5A, Kruskal-Wallis *p* < 0.001). In contrast to these findings, *p57*^*Kip2*^ mRNA was almost completely downregulated in metastases versus normal pancreas (Figure 5B, *p* < 0.01). *Menin* mRNA expression was not different in this pNEN sample set compared to normal tissue (Figure 5C). In addition, we identified a positive correlation between *NAP1L1* mRNA levels and *Ki67* (*r* = 0.64, *p* < 0.0001), *CgA* (*r* = 0.42, *p* < 0.001), *Chromogranin B* (*r* = 0.61, *p* < 0.0001), and the pNEN biomarker, *INA*[32] (*r* = 0.64, *p* < 0.0001) (*data not shown*). *NAP1L1* expression was also positively correlated with tumor size (Figure 5D, Spearman’s *r* = 0.4, *p* < 0.05). The mRNA results were confirmed on protein level (Figure 5E): NAP1L1 was over-expressed in pNEN primaries (*p* < 0.05) and pNEN metastasis (*p* < 0.05) compared to normal pancreas (Figure 5F,D), while p57^Kip2^ protein levels were higher in normal pancreas than in pNENs (*p* < 0.05, Figure 5G,D). Menin protein expression was also not different in this pNEN sample set compared to normal pancreatic tissue (Figure 5H,D).

## Discussion

The etiology and tumorigenesis of pancreatic NENs, which are the most aggressive form of gastroenteropancreatic NENs [38], remain poorly understood. Epigenetic modifications are unarguably one of the major mechanisms for pNEN pathogenesis [4]. In the present study, we examined the expression and function of chromatin modulator NAP1L1 in pancreatic NENs. While other NAP1 family members, e.g., NAP1L2, NAP1L3, and NAP1L5 [11-14] are well known to be neuron-specific and play a role in proliferation [15] and the NAP1 family is associated with transcriptional regulation [8], our current results define a role for NAP1L1 in the transcriptional regulation of pancreatic neuroendocrine neoplasm cell cycle progression. Specifically, our results highlight a role for this family member as an inhibitor of p57^Kip2^.

The current studies demonstrate (1) that NAP1L1 was over-expressed, while p57^Kip2^ was downregulated in pNENs, (2) that NAP1L1 is involved in the mTOR pathway signaling, (3) that NAP1L1 was essential for proliferation *in vitro* and in an orthotopic pNEN mouse model, and (4) that one mechanism of NAP1L1-mediated cell proliferation control may occur through inhibition of p57^Kip2^ transcription.

P57^Kip2^ has many functions; as it can arrest the cell cycle, promote apoptosis, and inhibit angiogenesis as well as its downregulation in many cancers [39], it qualifies as a tumor suppressor. It is also important in embryogenesis; *p57*^*Kip2*^-null mice exhibit high neonatal mortality with severe developmental defects [40]. In neoplasia, downregulation of p57^Kip2^ promotes cell migration, and invasion in human nasopharyngeal carcinoma cells [41] and decreased expression were associated with poor postsurgical survival time and lymphatic metastasis in lung cancer [42] as well as in other cancer types [25,39], e.g., in hepatocellular [43] or pancreatic [27] carcinoma. Our studies confirmed downregulation of p57^Kip2^ in pNENs and suggest one mechanism for this phenomenon. The promoter region of the *p57*^*Kip2*^ gene is rich in CpG islands and promoter methylation is a well-described mechanism to explain attenuated expression in cancers [39]. In a panel of tumor cell lines and primary cancer cells, dense *p57*^*Kip2*^ promoter CpG methylation was identified at the transcription start site [44]. We determined a role for NAP1L1 in the regulation of *p57*^*Kip2*^ promoter methylation in BON cells by direct promoter binding of NAP1L1. NAP1-like proteins have previously been reported to control gene expression through histone H3 acetylation [15]. Methylation, through a NAP1L1-complex, is therefore a novel mechanism for *p57*^*Kip2*^ transcriptional control that may be applicable to other neoplasia. This is supported by studies with demethylating agents (5-azacytidine or 5-aza-2′-deoxycytidine) which results in *p57*^*Kip2*^ re-expression or over-expression in several experimental cancer models [45,46]. Our observations suggest a direct link between NAP1L1 and p57^Kip2^. Targeting NAP1L1 (through silencing) increased the expression of p57^Kip2^ and resulted in significant decreases in both DNA synthesis as well as metastasis *in vivo*. Mechanistically, NAP1L1 bound to SP1/CTIP2 sites that are involved in HDAC-mediated transcriptional regulation of *p57*^*Kip2*^[37]. These sites are dissimilar to those identified for NAP1L2 binding in the promoter region in ES-derived differentiated neurons [15]. As NAP1L2 is associated with transcriptional repression through increased levels of acetylated histone H3K9/14, we conclude that NAP1L1 may inhibit transcription either by a similar mechanism or through inhibition of SP1-mediated activity [47].

The principal growth factor signaling pathway also affected by NAP1L1-silencing was the mTOR pathway. We identified that NAP1L1 knockdown both reduced phosphorylation of downstream mTOR effectors including 4E-BP1 as well as S6K1 and was associated with a decreased ribosomal RNA expression, all markers consistent with mTOR inhibition [35]. The ERK pathway, in contrast, was unaffected (Figure 1B). This suggests that NAP1L1 may promote proliferation via mTOR signaling. Since mutations of this pathway have been reported in 15% of pNENs [4], a role for NAP1L1 in this pathway may be of clinical relevance.

Menin, also a tumor suppressor, is one of the gene products predominantly mutated in pNENs [4,30]. The mechanism(s) of menin-driven tumor pathogenesis, however, remain unclear [2,48]. Menin can function as a histone-methyltransferase and is reported to have an anti-proliferative regulatory effect in pancreatic islet cells through histone H3K4 methylation and regulation of cell cycle inhibitors [49]. We anticipated that menin expression would be decreased in BON cells and clinical samples. We could not identify an altered expression in clinical samples or in the cell line (consistent with previous reports [50,51]). One reason may be the absence of *MEN1* mutations in our sample set. A second reason might be that despite a mutation of one menin allele, *menin* mRNA is detectable by PCR while the menin-antibody used (Cell Signaling Technology) recognizes all three isoforms of menin. This suggests that menin expression is not linked to NAP1L1-mediated effects. However, we did identify that menin protein expression was increased in NAP1L1 silenced cells which suggests that NAP1L1 may function to negatively regulate menin expression. This potentially identifies an additional role for NAP1L1 in pNEN pathogenesis.

## Conclusions

Our data identifies that NAP1L1 epigenetically promotes tumor cell proliferation in pancreatic neuroendocrine neoplasms through inhibition of the mTOR pathway and the tumor suppressor p57^Kip2^; the principal mechanism is via p57^Kip2^ promoter methylation. This turns NAP1L1 into a promising target for anti-cancer therapy.

## Methods

### Cell lines

To investigate mechanistic roles and the importance of NAP1L1 in proliferation, we used the adherent human metastasized pancreatic neuroendocrine neoplasm (NEN) cell line, BON [33], authenticated by STR (Genetica DNA Laboratories, Cincinnati, OH, USA). The cells were cultivated in medium containing RPMI 1640, Hams’ F12 (Gibco, Grand Island, NY, USA; 1:1 ratio), 10% FBS and penicillin/streptomycin (100 IU/ml) in 75 cm^2^ flasks (Sarstedt, Newton, NC, USA) as described [32].

### NAP1L1 knockdown

To assess the role of NAP1L1 in cell proliferation, we seeded 1.6 × 10^5^ BON cells/well in 6-well plates (Falcon, BD, Franklin Lakes, NJ, USA) and silenced NAP1L1 using reverse transfection with siRNA (sequence GGUAGAAACACCAACAGGAUACAUU, 140 pmol/well) and Lipofectamine RNAiMAX (Invitrogen, Carlsbad, CA, USA). We confirmed the knockdown using PCR and Western blot after 24–96 h. Control cells were treated with Lipofectamine and scrambled siRNA.

### Proliferation assay

To investigate the role of NAP1L1, p57^Kip2^, and menin in cell proliferation, we first cultured BON cells (2.5 × 10^5^/well) in 6-well plates (Falcon, BD, Franklin Lakes, NJ, USA) under normal conditions with (normal/logarithmic growth) and without serum (starvation/quiescent) and harvested them after 2 days for FACS (to determine the cell cycle kinetics of those BON cells, *seeFAC-sortingsection*) and protein isolation. In a second set of experiments, we used a BrdU (bromodeoxyuridine) chemiluminescence ELISA (Roche Diagnostics, Indianapolis, IN, USA) to investigate the proliferation of normal and NAP1L1-silenced BON cells according to the manufacturers’ instructions. Briefly, NAP1L1-silenced BON cells were labeled in 12-well plates (Falcon) with BrdU solution and incubated for 3 h. After fixation and DNA denaturation, BrdU incorporation, as a measurement of cell division, was quantitated using a chemiluminescence assay (GLOMAX Luminometer, Promega, Madison, WI, USA).

### Inhibition of the PI3K/mTOR and MAP-kinase pathway

We evaluated growth signaling and expression of *NAP1L1*, *p57*^*Kip2*^, and *menin* using selective pharmaceutical compounds that specifically inhibited growth-signaling-pathway kinases. We seeded BON cells (3 × 10^5^ cells/well) in 6-well plates (Falcon) for 24 h and treated these cells with GSK1120212 [MAPK pathway inhibitor (target MEK1/2) − 10^−7^ M) and BEZ235 [mTOR pathway inhibitor (target PI_3_K/PKB) − 10^−8^ M) for 24 h prior to mRNA collection. Cells were harvested by adding TRIZOL® (Invitrogen, Carlsbad, CA, USA) (*see Isolation of RNA and genomic DNA, reverse transcription, and quantitative PCRsection*).

### FAC-sorting

To quantify the cell cycle phases of BON cell after serum deprivation versus logarithmic growth (*see Proliferation assay section*), we harvested those BON cells and after 15 min incubation with propidium iodide/PBS (1:50, Roche, Indianapolis, IN, USA) conducted flow cytometry (BD FACS Aria Cell Sorter, BD Biosciences, Bedford, MA, USA). Cell cycle alterations were analyzed using FloJo Software 31 (TreeStar, Mountain View, CA, USA) [52].

### Orthotopic murine xenograft model

To assess the role of NAP1L1 in tumor cell proliferation *in vivo*, we attempted to establish a permanent NAP1L1 knockdown BON cell line by transfection of a NAP1L1-shRNA plasmid containing puromycin resistance gene and GFR luminescence (GeneCopoeia, Rockville, MD, USA) via Lipofectamine RNAiMAX (Invitrogen). Because proliferation of those cells was decreased so significantly following transfection (see Figure 2D), we could not culture the necessary number of cells for *in vivo* experiments. As an alternative, we grew BON cells in 75 cm^2^ flasks (Sarstedt) and transfected them in these flasks overnight in antibiotic-free medium. After confirming the internalization of the plasmid through fluorescence microscopy (488 nm), stable shRNA clones were selected via growth media containing 3 μg/ml puromycin (Invitrogen) and cells were harvested after 48 h (viability 77%–90%). We injected 1.5 × 10^6^ of the transfected cells or control BON cells (Lipofectamine treatment, viability >90%) in 100 μl PBS into the pancreas tail of nine Ns/J-mice (Jackson Laboratory, Bar Harbor, ME, USA) by flank-laparotomy under anesthesia [53]. All mice were euthanized 6 weeks after BON cell implantation, and circulating blood and tumor tissue were collected. All animals were treated according to IRB standards of the Yale University.

### Human sample collection

The collection of pancreatic tissue from patients with pNENs (*n* = 43) and normal pancreas (*n* = 10) was obtained according to the Ethics Committee requirements for the University of Heidelberg, Germany and an IRB protocol at Yale University [32]. Quality control was assured by a pathologist experienced with NEN histopathology. Only samples in which histology demonstrated more than 70% tumor were included. As only five patients had died, survival analysis was not undertaken.

### Protein extraction and western blot analysis

Tissue (1 × 2 mm) or cell line lysates were manually homogenized (PYREX homogenizer; VWR, Radnor, PA, USA) and prepared as previously described [32]. Supernatant protein was quantified (Pierce BCA protein assay kit; Thermo Scientific, Rockford, IL, USA), total protein lysates (15 μg) were denaturated in SDS sample buffer, separated on an SDS-PAGE gel [10% or 4%–20% (mTOR and 4E-BP1)], and transferred to a PVDF membrane (Bio-Rad, Hercules, CA, USA; pore size 0.45 mm). Primary antibodies included anti-NAP1L1 (Abcam, Cambridge, MA, USA), anti-p57^Kip2^, anti-menin-1, anti-phospho-ERK, anti-ERK, anti-phospho-mTOR, anti-mTOR, anti-phospho-S6K1, anti-S6K1, anti-phospho-4E-BP1, and anti-4E-BP1 (all from Cell Signaling Technology, Beverly, MA, USA). Immunodetection was performed using Western Lightning™ Plus-ECL (PerkinElmer, Waltham, MA, USA). Protein levels were confirmed with β-actin (Sigma-Aldrich, St. Louis, MO, USA). The optical density of the appropriately sized bands was measured using ImageJ software (NIH, Bethesda, MD, USA). The ratio between target protein expression and control protein was calculated.

### Isolation of RNA and genomic DNA, reverse transcription, and quantitative PCR

Messenger RNA was extracted from each tissue samples (or cell lines) using TRIZOL® (Invitrogen, Carlsbad, CA, USA) and cleaned (RNeasy kit, Qiagen, Valencia, CA, USA). After conversion to cDNA (high-capacity cDNA Archive Kit, Applied Biosystems, Carlsbad, CA, USA), quantitative PCR (qPCR) analyses were performed using Assays-on Demand™ and the ABI 7900 Sequence Detection System. Primer sets were all obtained from Applied Biosystems. PCR data was normalized using the ∆∆*C*_T_ method; *ALG9* was used as a housekeeping gene [54].

The extraction of genomic DNA was performed using the MasterPure Complete DNA and RNA Purification Kit (EPICENTRE, Madison, WI, USA) according to the manufacturers’ instructions and quantified by Nanodrop (Thermo Scientific).

### *p57*^*Kip2*^ promoter methylation

To investigate the status of *p57*^*Kip2*^ promoter methylation, we treated genomic DNA of normal BON cells and NAP1L1-silenced BON cells with bisulfate, which caused conversion of unmethylated cytosines into uracil. This was undertaken using the Methyl Code Bisulfite Conversion Kit (Invitrogen) according to the manufacturers’ instructions. MSPCR was performed using two primers which bound to the *p57*^*Kip2*^ promoter region (NCBI reference sequence NG_008022.1) (first forward primer, CGCCAATCGCCGTGGTGTTG [−164 to −141]; second forward primer, ACTACATTATGCTAATCGCG [−126 to −107]; reverse primer, GCCAGGCCTGAGCGAGCGAG) [+2 to +21]). The forward primers encompass SP1 and CTIP2 binding sites involved in HDAC-mediated transcriptional regulation [37]. Unmethylated promoter resulted in low PCR products in this assay.

### Chromatin immunoprecipitation

To examine whether NAP1L1 directly bound to the *p57*^*Kip2*^ promoter, we performed ChIP using the Pierce Agarose ChIP Kit (Thermo Scientific). Briefly, we fixed the crosslinks between DNA and protein by formaldehyde, lysed the cells, and digested chromatin using micrococcal nuclease. Of these DNA fragments, 10% was used as input control. After immunoprecipitation with a ChIP-grade-NAP1L1-antibody (Santa Cruz Biotechnology, Santa Cruz, CA, USA), DNA fragments were purified and qPCR performed using primers for detecting the *p57*^*Kip2*^ promoter (see above). We used normal rabbit IgG as negative and anti-RNA polymerase II antibody and GAPDH promoter primers as positive controls. The *p57*^*Kip2*^ promoter expression in NAP1L1-bound DNA fragments was calculated relative to the control.

### Statistical evaluation

All statistical analyses were performed using Microsoft Excel and Prism 5 (GraphPad Software, San Diego, CA, USA). Comparisons between more than two groups were performed using the Kruskal-Wallis test, followed by the Dunn’s *post hoc* test where appropriate. Binary comparisons were made using a 2-tailed Mann–Whitney *U* test. Correlations were undertaken using Spearman’s correlation. A *p* value of <0.05 was designated as significant. Statistical significance is indicated by an asterisk and described in the figure legends. Bars without an asterisk did not reach significance.

## Abbreviations

BrdU: bromdesoxyuridin; CgA: chromogranin A; ChIP: chromatin immunoprecipitation; HDAC: histone deacetylase; INA: internexin alpha; MEN-1: multiple endocrine neoplasia 1; mTOR: mechanistic (mammalian) target of rapamycin; NAP1L1: nucleosome assembly protein 1-like 1; NF-1: neurofibromatosis 1; pNENs: pancreatic neuroendocrine neoplasms; RPL28: ribosomal protein L28; RPS24: ribosomal protein S24; S6K1: S6 kinase 1; TSC: tuberous sclerosis complex; VHL: Von Hippel-Lindau disease; 4E-BP1: eukaryotic initiation factor 4E-binding protein.

## Competing interests

The authors declare that they have no competing interests.

## Authors’ contributions

MK, IM, and SS generated the main idea of the work and developed the study design, both conceptually and methodologically. AT and DA carried out methylation studies, isolation of mRNA and protein as well as Western blot and PCR. BL, MK, and SS contributed to analysis and interpretation of the data. HSW and MB contributed materials. SS and MK wrote the manuscript. BL, HSW, MB, and IM were involved in interpretation of data and critical revision for important intellectual content. All authors read and approved the final manuscript.
